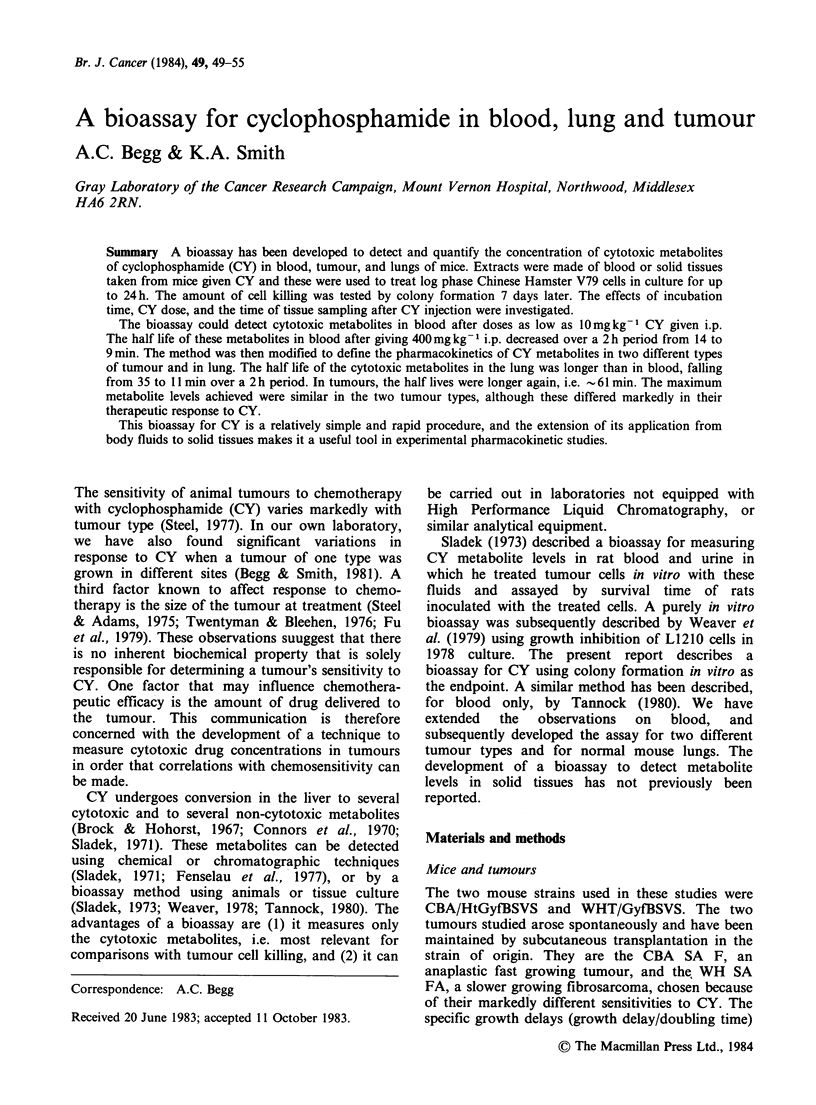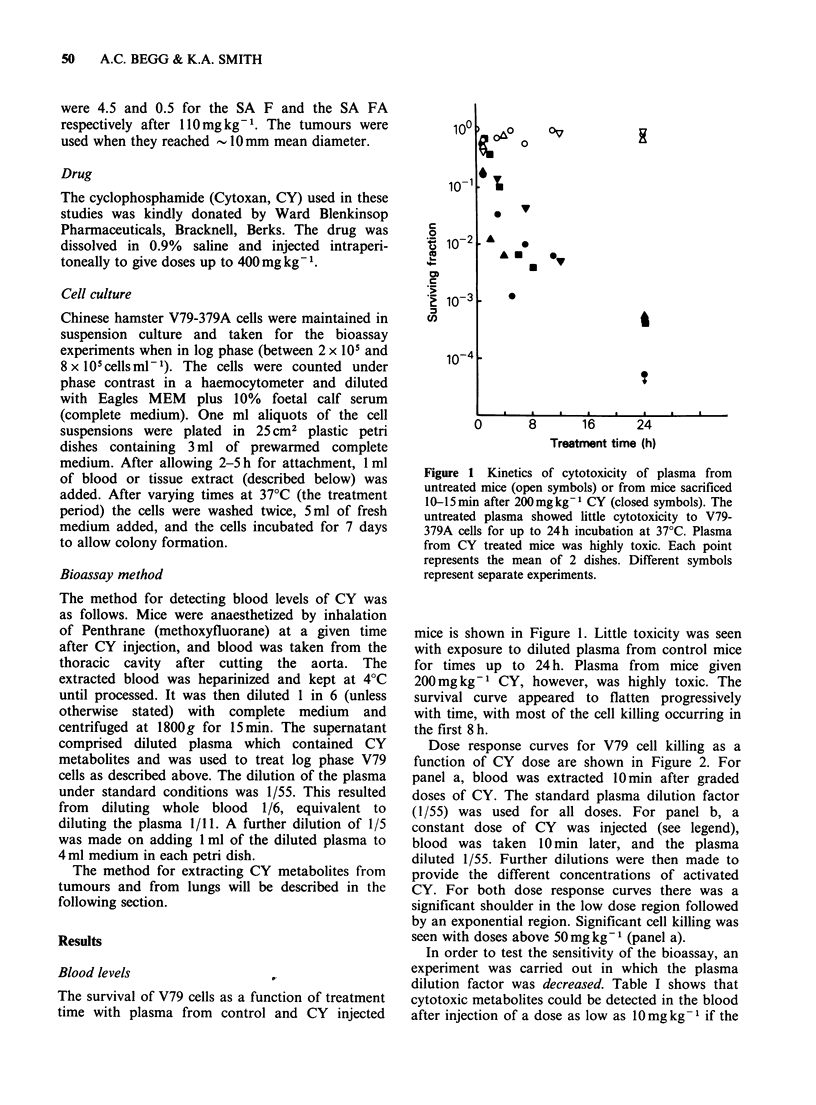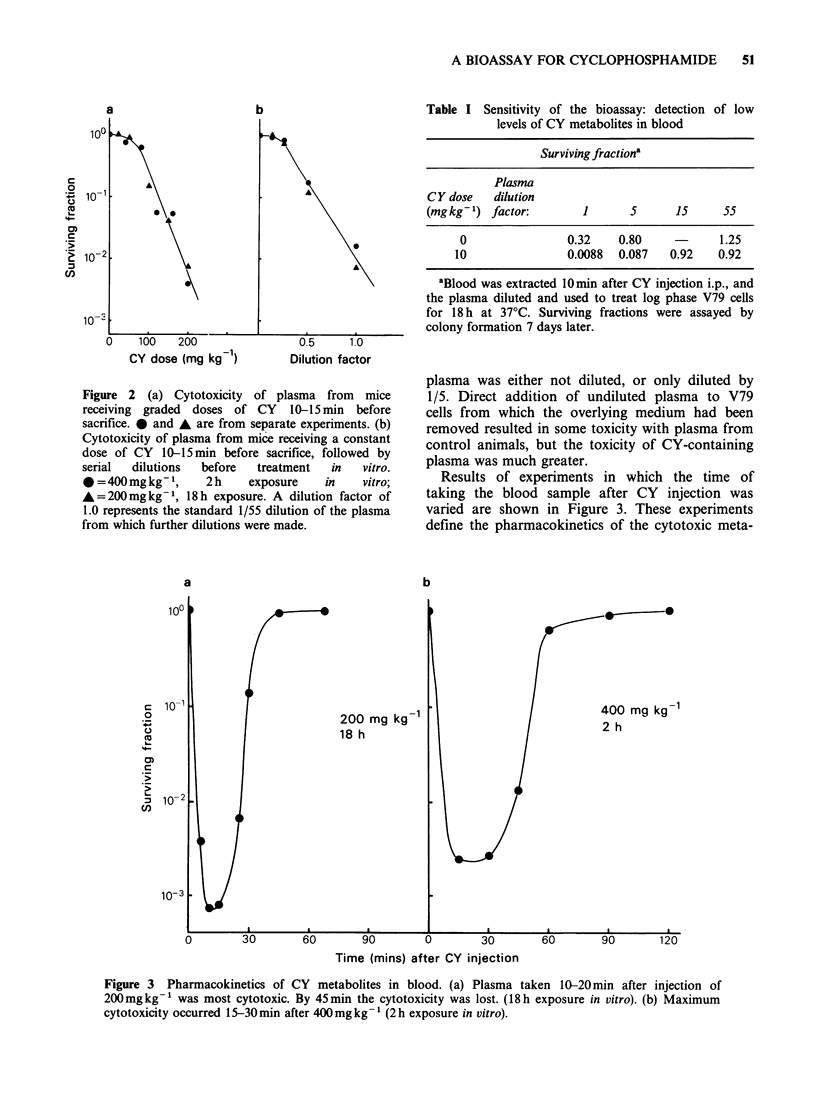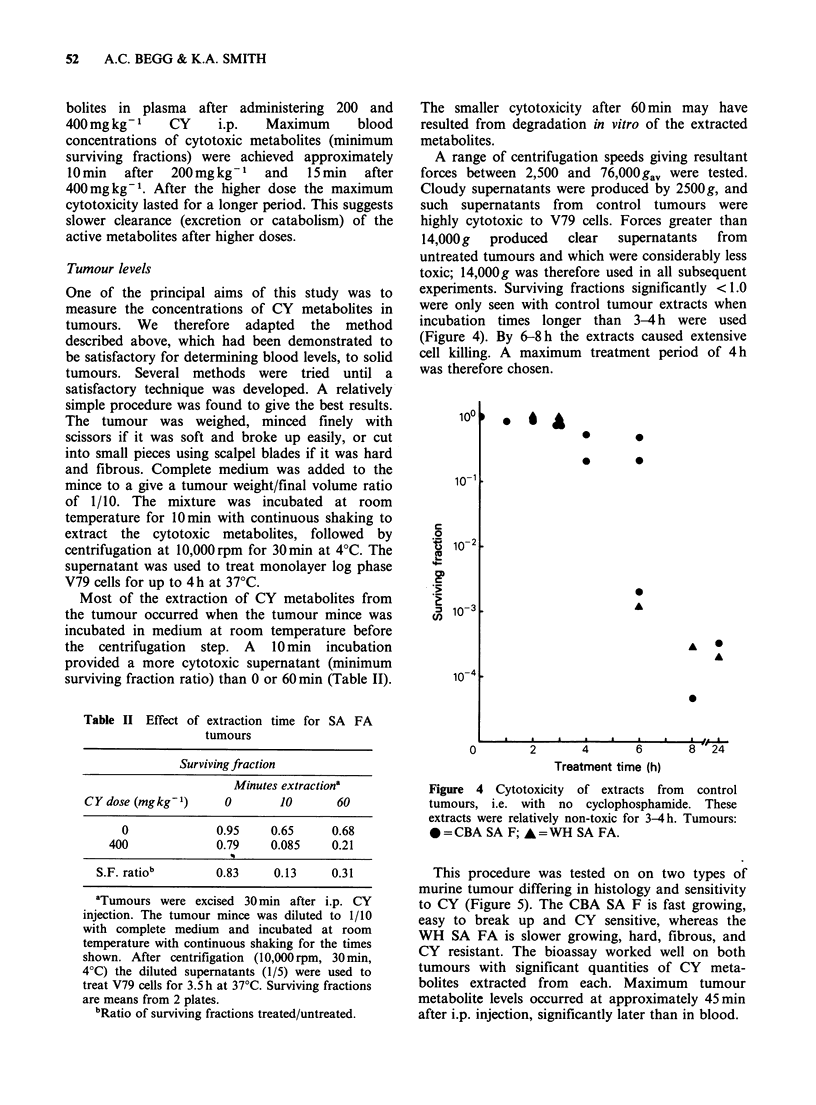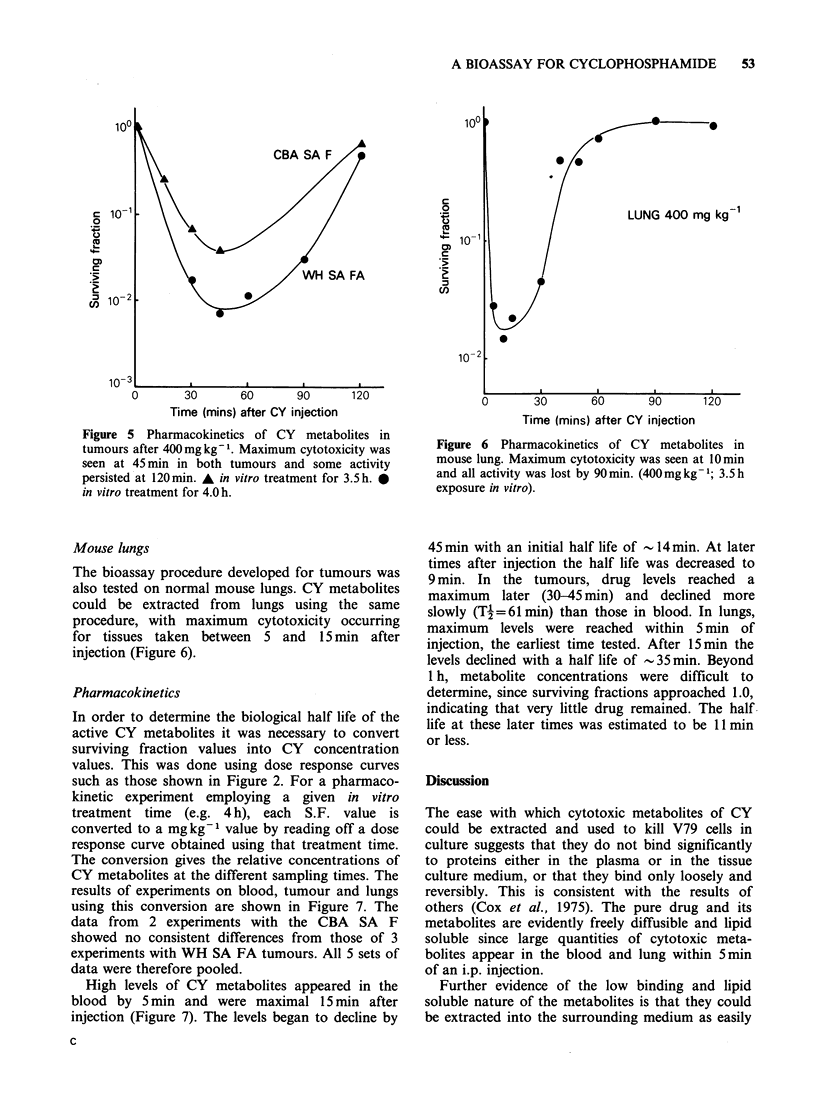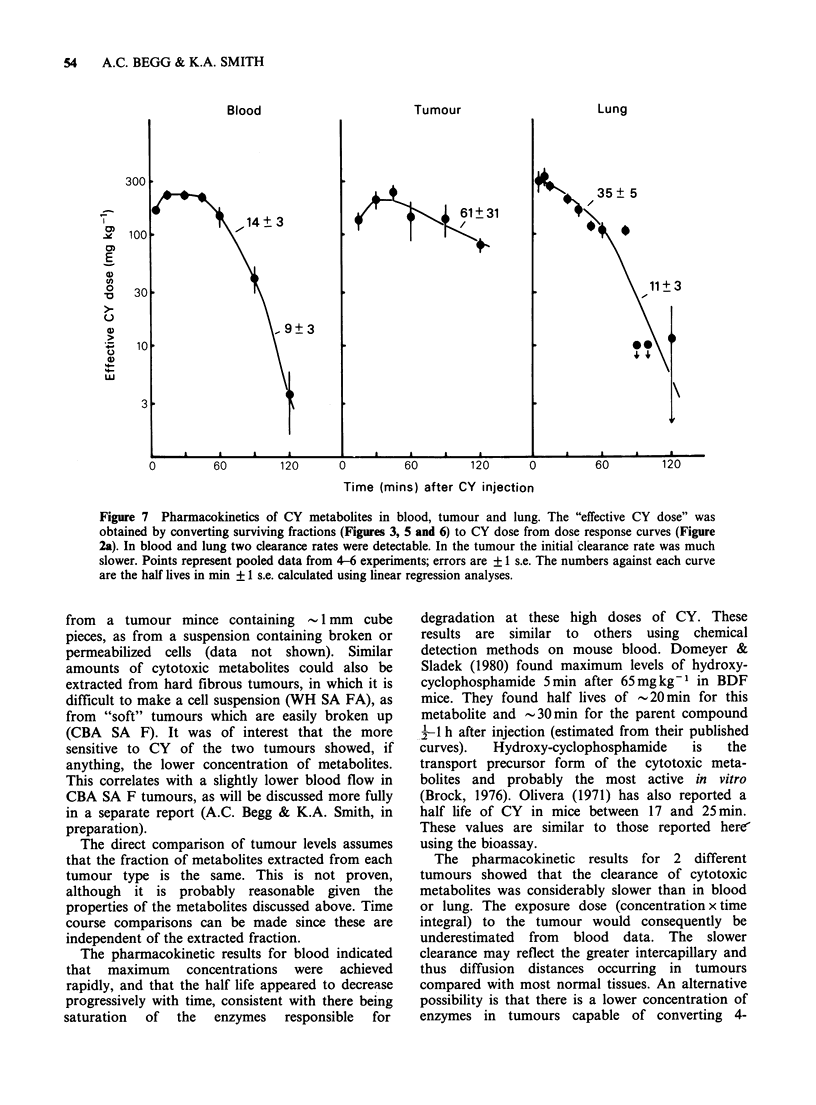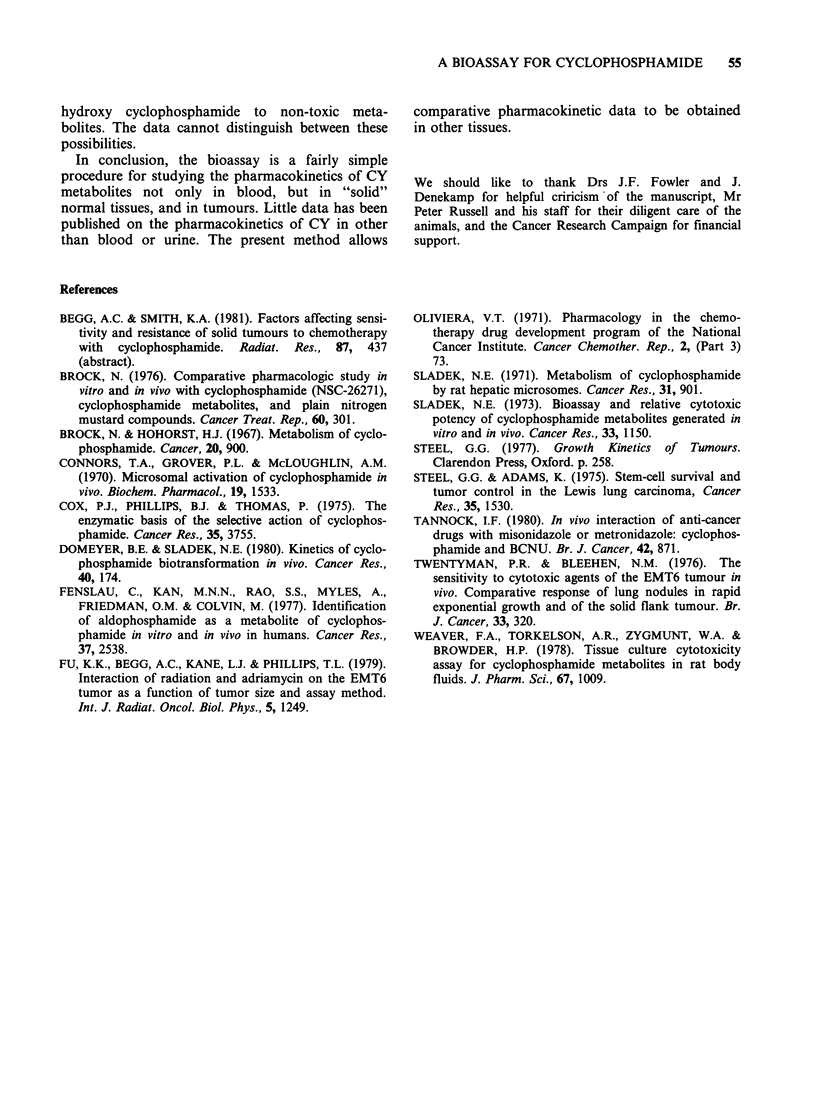# A bioassay for cyclophosphamide in blood, lung and tumour.

**DOI:** 10.1038/bjc.1984.8

**Published:** 1984-01

**Authors:** A. C. Begg, K. A. Smith

## Abstract

A bioassay has been developed to detect and quantify the concentration of cytotoxic metabolites of cyclophosphamide (CY) in blood, tumour, and lungs of mice. Extracts were made of blood or solid tissues taken from mice given CY and these were used to treat log phase Chinese Hamster V79 cells in culture for up to 24 h. The amount of cell killing was tested by colony formation 7 days later. The effects of incubation time, CY dose, and the time of tissue sampling after CY injection were investigated. The bioassay could detect cytotoxic metabolites in blood after doses as low as 10 mg kg-1 CY given i.p. The half life of these metabolites in blood after giving 400 mg kg-1 i.p. decreased over a 2 h period from 14 to 9 min. The method was then modified to define the pharmacokinetics of CY metabolites in two different types of tumour and in lung. The half life of the cytotoxic metabolites in the lung was longer than in blood, falling from 35 to 11 min over a 2 h period. In tumours, the half lives were longer again, i.e. approximately 61 min. The maximum metabolite levels achieved were similar in the two tumour types, although these differed markedly in their therapeutic response to CY. The bioassay for CY is a relatively simple and rapid procedure, and the extension of its application from body fluids to solid tissues makes it a useful tool in experimental pharmacokinetic studies.


*Br. J. Cancer* (1984), **49**, 49–55

### A bioassay for cyclophosphamide in blood, lung and tumour

A.C. Begg & K.A. Smith

*Gray Laboratory of the Cancer Research Campaign, Mount Vernon Hospital, Northwood, Middlesex HA6 2RN.*

**Summary** A bioassay has been developed to detect and quantify the concentration of cytotoxic metabolites of cyclophosphamide (CY) in blood, tumour, and lungs of mice. Extracts were made of blood or solid tissues taken from mice given CY and these were used to treat log phase Chinese Hamster V79 cells in culture for up to 24 h. The amount of cell killing was tested by colony formation 7 days later. The effects of incubation time, CY dose, and the time of tissue sampling after CY injection were investigated.

The bioassay could detect cytotoxic metabolites in blood after doses as low as $10\,mg\,kg^{-1}$ CY given i.p. The half life of these metabolites in blood after giving $400\,mg\,kg^{-1}$ i.p. decreased over a 2 h period from 14 to 9 min. The method was then modified to define the pharmacokinetics of CY metabolites in two different types of tumour and in lung. The half life of the cytotoxic metabolites in the lung was longer than in blood, falling from 35 to 11 min over a 2 h period. In tumours, the half lives were longer again, i.e. ~61 min. The maximum metabolite levels achieved were similar in the two tumour types, although these differed markedly in their therapeutic response to CY.

This bioassay for CY is a relatively simple and rapid procedure, and the extension of its application from body fluids to solid tissues makes it a useful tool in experimental pharmacokinetic studies.

The sensitivity of animal tumours to chemotherapy with cyclophosphamide (CY) varies markedly with tumour type (Steel, 1977). In our own laboratory, we have also found significant variations in response to CY when a tumour of one type was grown in different sites (Begg & Smith, 1981). A third factor known to affect response to chemotherapy is the size of the tumour at treatment (Steel & Adams, 1975; Twentyman & Bleehen, 1976; Fu *et al.*, 1979). These observations suugest that there is no inherent biochemical property that is solely responsible for determining a tumour's sensitivity to CY. One factor that may influence chemotherapeutic efficacy is the amount of drug delivered to the tumour. This communication is therefore concerned with the development of a technique to measure cytotoxic drug concentrations in tumours in order that correlations with chemosensitivity can be made.

CY undergoes conversion in the liver to several cytotoxic and to several non-cytotoxic metabolites (Brock & Hohorst, 1967; Connors *et al.*, 1970; Sladek, 1971). These metabolites can be detected using chemical or chromatographic techniques (Sladek, 1971; Fenselau *et al.*, 1977), or by a bioassay method using animals or tissue culture (Sladek, 1973; Weaver, 1978; Tannock, 1980). The advantages of a bioassay are (1) it measures only the cytotoxic metabolites, i.e. most relevant for comparisons with tumour cell killing, and (2) it can be carried out in laboratories not equipped with High Performance Liquid Chromatography, or similar analytical equipment.

Sladek (1973) described a bioassay for measuring CY metabolite levels in rat blood and urine in which he treated tumour cells *in vitro* with these fluids and assayed by survival time of rats inoculated with the treated cells. A purely *in vitro* bioassay was subsequently described by Weaver *et al.* (1979) using growth inhibition of L1210 cells in 1978 culture. The present report describes a bioassay for CY using colony formation *in vitro* as the endpoint. A similar method has been described, for blood only, by Tannock (1980). We have extended the observations on blood, and subsequently developed the assay for two different tumour types and for normal mouse lungs. The development of a bioassay to detect metabolite levels in solid tissues has not previously been reported.

### Materials and methods

### *Mice and tumours*

The two mouse strains used in these studies were CBA/HtGyfBSVS and WHT/GyfBSVS. The two tumours studied arose spontaneously and have been maintained by subcutaneous transplantation in the strain of origin. They are the CBA SA F, an anaplastic fast growing tumour, and the WH SA FA, a slower growing fibrosarcoma, chosen because of their markedly different sensitivities to CY. The specific growth delays (growth delay/doubling time)

Correspondence: A.C. Begg

Received 20 June 1983; accepted 11 October 1983.

© The Macmillan Press Ltd., 1984

were 4.5 and 0.5 for the SA F and the SA FA respectively after 110 mg kg$^{-1}$. The tumours were used when they reached ~10 mm mean diameter.

*Drug*

The cyclophosphamide (Cytoxan, CY) used in these studies was kindly donated by Ward Blenkinsop Pharmaceuticals, Bracknell, Berks. The drug was dissolved in 0.9% saline and injected intraperitoneally to give doses up to 400 mg kg$^{-1}$.

*Cell culture*

Chinese hamster V79-379A cells were maintained in suspension culture and taken for the bioassay experiments when in log phase (between $2 \times 10^5$ and $8 \times 10^5$ cells ml$^{-1}$). The cells were counted under phase contrast in a haemocytometer and diluted with Eagles MEM plus 10% foetal calf serum (complete medium). One ml aliquots of the cell suspensions were plated in 25 cm$^2$ plastic petri dishes containing 3 ml of prewarmed complete medium. After allowing 2–5 h for attachment, 1 ml of blood or tissue extract (described below) was added. After varying times at 37°C (the treatment period) the cells were washed twice, 5 ml of fresh medium added, and the cells incubated for 7 days to allow colony formation.

*Bioassay method*

The method for detecting blood levels of CY was as follows. Mice were anaesthetized by inhalation of Penthrane (methoxyfluorane) at a given time after CY injection, and blood was taken from the thoracic cavity after cutting the aorta. The extracted blood was heparinized and kept at 4°C until processed. It was then diluted 1 in 6 (unless otherwise stated) with complete medium and centrifuged at 1800 *g* for 15 min. The supernatant comprised diluted plasma which contained CY metabolites and was used to treat log phase V79 cells as described above. The dilution of the plasma under standard conditions was 1/55. This resulted from diluting whole blood 1/6, equivalent to diluting the plasma 1/11. A further dilution of 1/5 was made on adding 1 ml of the diluted plasma to 4 ml medium in each petri dish.

The method for extracting CY metabolites from tumours and from lungs will be described in the following section.

### Results

*Blood levels*

The survival of V79 cells as a function of treatment time with plasma from control and CY injected mice is shown in Figure 1. Little toxicity was seen with exposure to diluted plasma from control mice for times up to 24 h. Plasma from mice given 200 mg kg$^{-1}$ CY, however, was highly toxic. The survival curve appeared to flatten progressively with time, with most of the cell killing occurring in the first 8 h.

**Figure 1** Kinetics of cytotoxicity of plasma from untreated mice (open symbols) or from mice sacrificed 10–15 min after 200 mg kg$^{-1}$ CY (closed symbols). The untreated plasma showed little cytotoxicity to V79-379A cells for up to 24 h incubation at 37°C. Plasma from CY treated mice was highly toxic. Each point represents the mean of 2 dishes. Different symbols represent separate experiments.

Dose response curves for V79 cell killing as a function of CY dose are shown in Figure 2. For panel a, blood was extracted 10 min after graded doses of CY. The standard plasma dilution factor (1/55) was used for all doses. For panel b, a constant dose of CY was injected (see legend), blood was taken 10 min later, and the plasma diluted 1/55. Further dilutions were then made to provide the different concentrations of activated CY. For both dose response curves there was a significant shoulder in the low dose region followed by an exponential region. Significant cell killing was seen with doses above 50 mg kg$^{-1}$ (panel a).

In order to test the sensitivity of the bioassay, an experiment was carried out in which the plasma dilution factor was *decreased*. Table I shows that cytotoxic metabolites could be detected in the blood after injection of a dose as low as 10 mg kg$^{-1}$ if the

**Figure 2** (a) Cytotoxicity of plasma from mice receiving graded doses of CY 10–15 min before sacrifice. ● and ▲ are from separate experiments. (b) Cytotoxicity of plasma from mice receiving a constant dose of CY 10–15 min before sacrifice, followed by serial dilutions before treatment *in vitro*. ● = 400 mg kg$^{-1}$, 2 h exposure *in vitro*; ▲ = 200 mg kg$^{-1}$, 18 h exposure. A dilution factor of 1.0 represents the standard 1/55 dilution of the plasma from which further dilutions were made.

**Table I** Sensitivity of the bioassay: detection of low levels of CY metabolites in blood

| | *Surviving fraction*[a] | | | |
|---|---|---|---|---|
| *CY dose (mg kg$^{-1}$)* / *Plasma dilution factor:* | *1* | *5* | *15* | *55* |
| 0 | 0.32 | 0.80 | — | 1.25 |
| 10 | 0.0088 | 0.087 | 0.92 | 0.92 |

[a]Blood was extracted 10 min after CY injection i.p., and the plasma diluted and used to treat log phase V79 cells for 18 h at 37°C. Surviving fractions were assayed by colony formation 7 days later.

plasma was either not diluted, or only diluted by 1/5. Direct addition of undiluted plasma to V79 cells from which the overlying medium had been removed resulted in some toxicity with plasma from control animals, but the toxicity of CY-containing plasma was much greater.

Results of experiments in which the time of taking the blood sample after CY injection was varied are shown in Figure 3. These experiments define the pharmacokinetics of the cytotoxic meta-

**Figure 3** Pharmacokinetics of CY metabolites in blood. (a) Plasma taken 10–20 min after injection of 200 mg kg$^{-1}$ was most cytotoxic. By 45 min the cytotoxicity was lost. (18 h exposure *in vitro*). (b) Maximum cytotoxicity occurred 15–30 min after 400 mg kg$^{-1}$ (2 h exposure *in vitro*).

bolites in plasma after administering 200 and 400 mg kg$^{-1}$ CY i.p. Maximum blood concentrations of cytotoxic metabolites (minimum surviving fractions) were achieved approximately 10 min after 200 mg kg$^{-1}$ and 15 min after 400 mg kg$^{-1}$. After the higher dose the maximum cytotoxicity lasted for a longer period. This suggests slower clearance (excretion or catabolism) of the active metabolites after higher doses.

*Tumour levels*

One of the principal aims of this study was to measure the concentrations of CY metabolites in tumours. We therefore adapted the method described above, which had been demonstrated to be satisfactory for determining blood levels, to solid tumours. Several methods were tried until a satisfactory technique was developed. A relatively simple procedure was found to give the best results. The tumour was weighed, minced finely with scissors if it was soft and broke up easily, or cut into small pieces using scalpel blades if it was hard and fibrous. Complete medium was added to the mince to a give a tumour weight/final volume ratio of 1/10. The mixture was incubated at room temperature for 10 min with continuous shaking to extract the cytotoxic metabolites, followed by centrifugation at 10,000 rpm for 30 min at 4°C. The supernatant was used to treat monolayer log phase V79 cells for up to 4 h at 37°C.

Most of the extraction of CY metabolites from the tumour occurred when the tumour mince was incubated in medium at room temperature before the centrifugation step. A 10 min incubation provided a more cytotoxic supernatant (minimum surviving fraction ratio) than 0 or 60 min (Table II).

**Table II** Effect of extraction time for SA FA tumours

| *Surviving fraction* | | | |
|---|---|---|---|
| | *Minutes extraction*[a] | | |
| *CY dose* (*mg kg*$^{-1}$) | *0* | *10* | *60* |
| 0 | 0.95 | 0.65 | 0.68 |
| 400 | 0.79 | 0.085 | 0.21 |
| S.F. ratio[b] | 0.83 | 0.13 | 0.31 |

[a]Tumours were excised 30 min after i.p. CY injection. The tumour mince was diluted to 1/10 with complete medium and incubated at room temperature with continuous shaking for the times shown. After centrifigation (10,000 rpm, 30 min, 4°C) the diluted supernatants (1/5) were used to treat V79 cells for 3.5 h at 37°C. Surviving fractions are means from 2 plates.

[b]Ratio of surviving fractions treated/untreated.

The smaller cytotoxicity after 60 min may have resulted from degradation *in vitro* of the extracted metabolites.

A range of centrifugation speeds giving resultant forces between 2,500 and 76,000 $g_{av}$ were tested. Cloudy supernatants were produced by 2500 *g*, and such supernatants from control tumours were highly cytotoxic to V79 cells. Forces greater than 14,000 *g* produced clear supernatants from untreated tumours and which were considerably less toxic; 14,000 *g* was therefore used in all subsequent experiments. Surviving fractions significantly $<1.0$ were only seen with control tumour extracts when incubation times longer than 3–4 h were used (Figure 4). By 6–8 h the extracts caused extensive cell killing. A maximum treatment period of 4 h was therefore chosen.

**Figure 4** Cytotoxicity of extracts from control tumours, i.e. with no cyclophosphamide. These extracts were relatively non-toxic for 3–4 h. Tumours: ● = CBA SA F; ▲ = WH SA FA.

This procedure was tested on on two types of murine tumour differing in histology and sensitivity to CY (Figure 5). The CBA SA F is fast growing, easy to break up and CY sensitive, whereas the WH SA FA is slower growing, hard, fibrous, and CY resistant. The bioassay worked well on both tumours with significant quantities of CY metabolites extracted from each. Maximum tumour metabolite levels occurred at approximately 45 min after i.p. injection, significantly later than in blood.

**Figure 5** Pharmacokinetics of CY metabolites in tumours after 400 mg kg$^{-1}$. Maximum cytotoxicity was seen at 45 min in both tumours and some activity persisted at 120 min. ▲ *in vitro* treatment for 3.5 h. ● *in vitro* treatment for 4.0 h.

**Figure 6** Pharmacokinetics of CY metabolites in mouse lung. Maximum cytotoxicity was seen at 10 min and all activity was lost by 90 min. (400 mg kg$^{-1}$; 3.5 h exposure *in vitro*).

*Mouse lungs*

The bioassay procedure developed for tumours was also tested on normal mouse lungs. CY metabolites could be extracted from lungs using the same procedure, with maximum cytotoxicity occurring for tissues taken between 5 and 15 min after injection (Figure 6).

*Pharmacokinetics*

In order to determine the biological half life of the active CY metabolites it was necessary to convert surviving fraction values into CY concentration values. This was done using dose response curves such as those shown in Figure 2. For a pharmacokinetic experiment employing a given *in vitro* treatment time (e.g. 4 h), each S.F. value is converted to a mg kg$^{-1}$ value by reading off a dose response curve obtained using that treatment time. The conversion gives the relative concentrations of CY metabolites at the different sampling times. The results of experiments on blood, tumour and lungs using this conversion are shown in Figure 7. The data from 2 experiments with the CBA SA F showed no consistent differences from those of 3 experiments with WH SA FA tumours. All 5 sets of data were therefore pooled.

High levels of CY metabolites appeared in the blood by 5 min and were maximal 15 min after injection (Figure 7). The levels began to decline by 45 min with an initial half life of ~14 min. At later times after injection the half life was decreased to 9 min. In the tumours, drug levels reached a maximum later (30–45 min) and declined more slowly ($T\frac{1}{2}$ = 61 min) than those in blood. In lungs, maximum levels were reached within 5 min of injection, the earliest time tested. After 15 min the levels declined with a half life of ~35 min. Beyond 1 h, metabolite concentrations were difficult to determine, since surviving fractions approached 1.0, indicating that very little drug remained. The half life at these later times was estimated to be 11 min or less.

### Discussion

The ease with which cytotoxic metabolites of CY could be extracted and used to kill V79 cells in culture suggests that they do not bind significantly to proteins either in the plasma or in the tissue culture medium, or that they bind only loosely and reversibly. This is consistent with the results of others (Cox *et al.*, 1975). The pure drug and its metabolites are evidently freely diffusible and lipid soluble since large quantities of cytotoxic metabolites appear in the blood and lung within 5 min of an i.p. injection.

Further evidence of the low binding and lipid soluble nature of the metabolites is that they could be extracted into the surrounding medium as easily

**Figure 7** Pharmacokinetics of CY metabolites in blood, tumour and lung. The "effective CY dose" was obtained by converting surviving fractions (**Figures 3, 5 and 6**) to CY dose from dose response curves (**Figure 2a**). In blood and lung two clearance rates were detectable. In the tumour the initial clearance rate was much slower. Points represent pooled data from 4–6 experiments; errors are $\pm 1$ s.e. The numbers against each curve are the half lives in min $\pm 1$ s.e. calculated using linear regression analyses.

from a tumour mince containing ~1 mm cube pieces, as from a suspension containing broken or permeabilized cells (data not shown). Similar amounts of cytotoxic metabolites could also be extracted from hard fibrous tumours, in which it is difficult to make a cell suspension (WH SA FA), as from "soft" tumours which are easily broken up (CBA SA F). It was of interest that the more sensitive to CY of the two tumours showed, if anything, the lower concentration of metabolites. This correlates with a slightly lower blood flow in CBA SA F tumours, as will be discussed more fully in a separate report (A.C. Begg & K.A. Smith, in preparation).

The direct comparison of tumour levels assumes that the fraction of metabolites extracted from each tumour type is the same. This is not proven, although it is probably reasonable given the properties of the metabolites discussed above. Time course comparisons can be made since these are independent of the extracted fraction.

The pharmacokinetic results for blood indicated that maximum concentrations were achieved rapidly, and that the half life appeared to decrease progressively with time, consistent with there being saturation of the enzymes responsible for degradation at these high doses of CY. These results are similar to others using chemical detection methods on mouse blood. Domeyer & Sladek (1980) found maximum levels of hydroxy-cyclophosphamide 5 min after 65 mg $kg^{-1}$ in BDF mice. They found half lives of ~20 min for this metabolite and ~30 min for the parent compound $\frac{1}{2}$–1 h after injection (estimated from their published curves). Hydroxy-cyclophosphamide is the transport precursor form of the cytotoxic metabolites and probably the most active *in vitro* (Brock, 1976). Olivera (1971) has also reported a half life of CY in mice between 17 and 25 min. These values are similar to those reported here using the bioassay.

The pharmacokinetic results for 2 different tumours showed that the clearance of cytotoxic metabolites was considerably slower than in blood or lung. The exposure dose (concentration × time integral) to the tumour would consequently be underestimated from blood data. The slower clearance may reflect the greater intercapillary and thus diffusion distances occurring in tumours compared with most normal tissues. An alternative possibility is that there is a lower concentration of enzymes in tumours capable of converting 4-

hydroxy cyclophosphamide to non-toxic metabolites. The data cannot distinguish between these possibilities.

In conclusion, the bioassay is a fairly simple procedure for studying the pharmacokinetics of CY metabolites not only in blood, but in "solid" normal tissues, and in tumours. Little data has been published on the pharmacokinetics of CY in other than blood or urine. The present method allows comparative pharmacokinetic data to be obtained in other tissues.

We should like to thank Drs J.F. Fowler and J. Denekamp for helpful criricism of the manuscript, Mr Peter Russell and his staff for their diligent care of the animals, and the Cancer Research Campaign for financial support.

### References

BEGG, A.C. & SMITH, K.A. (1981). Factors affecting sensitivity and resistance of solid tumours to chemotherapy with cyclophosphamide. *Radiat. Res.*, **87**, 437 (abstract).

BROCK, N. (1976). Comparative pharmacologic study *in vitro* and *in vivo* with cyclophosphamide (NSC-26271), cyclophosphamide metabolites, and plain nitrogen mustard compounds. *Cancer Treat. Rep.*, **60**, 301.

BROCK, N. & HOHORST, H.J. (1967). Metabolism of cyclophosphamide. *Cancer*, **20**, 900.

CONNORS, T.A., GROVER, P.L. & McLOUGHLIN, A.M. (1970). Microsomal activation of cyclophosphamide *in vivo*. *Biochem. Pharmacol.*, **19**, 1533.

COX, P.J., PHILLIPS, B.J. & THOMAS, P. (1975). The enzymatic basis of the selective action of cyclophosphamide. *Cancer Res.*, **35**, 3755.

DOMEYER, B.E. & SLADEK, N.E. (1980). Kinetics of cyclophosphamide biotransformation *in vivo*. *Cancer Res.*, **40**, 174.

FENSLAU, C., KAN, M.N.N., RAO, S.S., MYLES, A., FRIEDMAN, O.M. & COLVIN, M. (1977). Identification of aldophosphamide as a metabolite of cyclophosphamide *in vitro* and *in vivo* in humans. *Cancer Res.*, **37**, 2538.

FU, K.K., BEGG, A.C., KANE, L.J. & PHILLIPS, T.L. (1979). Interaction of radiation and adriamycin on the EMT6 tumor as a function of tumor size and assay method. *Int. J. Radiat. Oncol. Biol. Phys.*, **5**, 1249.

OLIVIERA, V.T. (1971). Pharmacology in the chemotherapy drug development program of the National Cancer Institute. *Cancer Chemother. Rep.*, **2**, (Part 3) 73.

SLADEK, N.E. (1971). Metabolism of cyclophosphamide by rat hepatic microsomes. *Cancer Res.*, **31**, 901.

SLADEK, N.E. (1973). Bioassay and relative cytotoxic potency of cyclophosphamide metabolites generated *in vitro* and *in vivo*. *Cancer Res.*, **33**, 1150.

STEEL, G.G. (1977). *Growth Kinetics of Tumours*. Clarendon Press, Oxford. p. 258.

STEEL, G.G. & ADAMS, K. (1975). Stem-cell survival and tumor control in the Lewis lung carcinoma, *Cancer Res.*, **35**, 1530.

TANNOCK, I.F. (1980). *In vivo* interaction of anti-cancer drugs with misonidazole or metronidazole: cyclophosphamide and BCNU. *Br. J. Cancer*, **42**, 871.

TWENTYMAN, P.R. & BLEEHEN, N.M. (1976). The sensitivity to cytotoxic agents of the EMT6 tumour *in vivo*. Comparative response of lung nodules in rapid exponential growth and of the solid flank tumour. *Br. J. Cancer*, **33**, 320.

WEAVER, F.A., TORKELSON, A.R., ZYGMUNT, W.A. & BROWDER, H.P. (1978). Tissue culture cytotoxicity assay for cyclophosphamide metabolites in rat body fluids. *J. Pharm. Sci.*, **67**, 1009.